# Mesenchymal Stem Cells Attenuated Blood-Brain Barrier Disruption via Downregulation of Aquaporin-4 Expression in EAE Mice

**DOI:** 10.1007/s12035-020-01998-z

**Published:** 2020-07-01

**Authors:** Yanqun Liu, Yuanyuan Ma, Bingying Du, Yongting Wang, Guo-Yuan Yang, Xiaoying Bi

**Affiliations:** 1 Department of Neurology, Shanghai Changhai Hospital, Second Military Medical University, 168 Changhai Road, Shanghai, 200433 China; 2 Neuroscience and Neuroengineering Research Center, Med-X Research Institute and School of Biomedical Engineering, Shanghai Jiao Tong University, Shanghai, 200030 China; 3 Department of Neurology, Zhongshan Hospital, Fudan University, Shanghai, 200032 China; 4 Department of Neurology, General Hospital of Central Theater Command of Chinese People’s Liberation Army, Wuhan, 430070 China

**Keywords:** Aquaporin-4, Blood-brain barrier, Experimental autoimmune encephalomyelitis, Mesenchymal stem cells, Multiple sclerosis

## Abstract

Blood-brain barrier disruption is one of the hallmarks of multiple sclerosis. Mesenchymal stem cells showed great potential for the multiple sclerosis therapy. However, the effect of mesenchymal stem cells on blood-brain barrier in multiple sclerosis remains unclear. Here, we investigated whether mesenchymal stem cells transplantation protected blood-brain barrier integrity and further explored possible underlying mechanisms. Adult female C57BL/6 mice were immunized with myelin oligodendrocyte glycoprotein peptide33-55 (MOG33-55) to induce experimental autoimmune encephalomyelitis (EAE). Mesenchymal stem cells (5 × 10^5^) were transplanted via tail vein at disease onset. In the cell culture, we examined lipopolysaccharide-induced AQP4 upregulation in astrocytes. Results indicated that mesenchymal stem cells therapy improved neurobehavioral outcomes in EAE mice, reduced inflammatory cell infiltration, IgG protein leakage, and demyelination in spinal cord. Mesenchymal stem cells therapy also increased tight junction protein expression. In addition, mesenchymal stem cells downregulated AQP4 and A_2B_ adenosine receptor (A_2B_AR) expression in EAE mice in spinal cord. We found that MSCs-conditioned medium (MCM) reduced the expression of inflammatory cytokines, AQP4 and A_2B_AR in lipopolysaccharide-activated astrocytes. BAY-60-6583 (a selective A_2B_AR agonist) reversed the MCM-induced AQP4 downregulation and increased p38 MAPK phosphorylation. Furthermore, the upregulation effects of A_2B_AR agonist were eliminated when treated with p38 MAPK inhibitor SB203580. Thus, we concluded that mesenchymal stem cells alleviated blood-brain barrier disruption by downregulating AQP4 in multiple sclerosis, possibly through inhibiting the A_2B_AR/p38 MAPK signaling pathway. Our work suggests that mesenchymal stem cells exert beneficial effect through maintaining blood-brain barrier integrity in EAE mice.

## Background

Multiple sclerosis (MS) is the most common inflammatory demyelinating disease of the central nervous system. It affects predominantly young adults [1]. Although a variety of treatment strategies are currently available for MS patients, none of these strategies can halt disease progression, and their side effects lead to poor adherence [2]. Therefore, developing effective treatment with greater efficacy and fewer adverse effects is still an imperative task.

The loss of blood-brain barrier (BBB) integrity is a hallmark of MS, and its occurrence precedes lesion formation in MS patients [3]. Disruption of the BBB promotes immune cell infiltration and impacts clinical outcomes [4, 5]. Therapeutics that stabilize BBB function is beneficial in MS. In experimental autoimmune encephalomyelitis (EAE) mice, enhanced expression of claudin-1 significantly reduced BBB leakiness and disease burden during the chronic phase [6]. The increased peroxiredoxin 6 (PRDX6) expression in astrocytes also alleviated clinical score in EAE mice [7]. Plasma kallikrein inhibition decreased BBB damage and cell invasion during neuroinflammation [8]. S1PR2 knockout decreased BBB leakage, the extent of demyelinated area, and the clinical disability [9]. Thus, protecting and restoring BBB function is a promising target of MS therapies.

Studies have demonstrated that mesenchymal stem cells (MSCs) is an attractive candidate for the treatment of MS [2]. MSCs improved the chronic progressive course of EAE, and this therapeutic effect was induced by T cell tolerance [10]. After MSCs treatment, Th1 cells and Th17 cells and their associated cytokines were reduced, while Th2 cells and anti-inflammatory cytokines were increased in EAE mice [11]. In addition, MSCs improved recovery and reduced relapse rate in relapsing-remitting model of EAE. The effect was mediated by inhibiting B cell infiltration into the central nervous system (CNS) and decreasing the production of pathogenic antibodies against myelin [12]. Apart from the immunomodulation effects of MSCs, studies demonstrated that MSCs transplantation promoted function recovery through the release of trophic factors. Conditioned medium from human MSCs reduced functional deficits in EAE mice and promoted the repairing and remodeling of oligodendrocytes and neurons. Hepatocyte growth factor (HGF) secreted by MSCs was responsible for the beneficial effects of MSCs [13]. Other neurotrophic factors, such as nerve growth factor (NGF), brain-derived neurotrophic factor (BDNF), and glial cell derived neurotrophic factor (GDNF), which were released by MSCs have also been demonstrated useful in promoting function recovery in MS [14, 15]. Consequently, MSCs transplantation not only alleviated function disability through immune-regulating ability, but also promoted function recovery through the release of trophic factors.

The protective effects of MSCs were visible only when MSCs was injected before or at the disease onset [10, 16]. which indicated that it might has the potential of maintaining BBB integrity and/or reducing the extravasation of immune cells, since they are among the earliest events in the pathogenesis of MS [3, 17]. Indeed, the beneficial effects of MSCs on BBB integrity have been investigated in several diseases. In ischemic stroke, MSCs transplantation maintained BBB integrity through inhibiting aquaporin-4 (AQP4) upregulation [18] and attenuating the upward trend of MMP9 [19]. MSCs decreased the degree of BBB leakage and improved neurological recovery in a rat intracerebral hemorrhage model by increasing TNF-stimulated gene/protein 6 (TSG-6) [20]. Administration of MSCs after transient global cerebral ischemia showed neuroprotective effects via preventing BBB disruption [21]. In LPS-induced inflamed brain, MSCs stabilized BBB permeability through modulating astrocytic end-feet and VEGF-A signaling [22]. MSCs reversed TNF-α-induced changes in tight junction protein and permeability in BBB model in vitro [23]. However, in MS, the effect of MSCs on the integrity of BBB is scarcely explored.

AQP4, expressed in the end-feet of the astrocyte, is a water channel protein. It is involved in brain water balance, neuroinflammation, neuroexcitation, and astrocyte migration [24]. Previous studies demonstrated that AQP4 expression was increased in both human MS lesion and EAE mice [25, 26]. AQP4 deficiency reduced neuroinflammation and resulted in milder clinical behavior in EAE mice [26, 27]. Increased AQP4 in astrocytes lead to cell edema, apoptosis of astrocytes, and disruption of the BBB [18, 24]. Reducing AQP4 expression can alleviate BBB disruption [18, 28]. Thus, AQP4 is a potential target in the treatment of MS.

Previous studies on the effects of MSCs were focusing on the immune-regulation and repair promotion function, but effects of MSCs on AQP4 expression and BBB maintenance in MS are still unknown. In our research, we explored whether MSCs could downregulate AQP4 expression and maintain BBB integrity in EAE mice. We attempted to investigate the underlying mechanism of MSCs treatment.

## Material and Methods

### Experimental Design

**Fig. 1:**
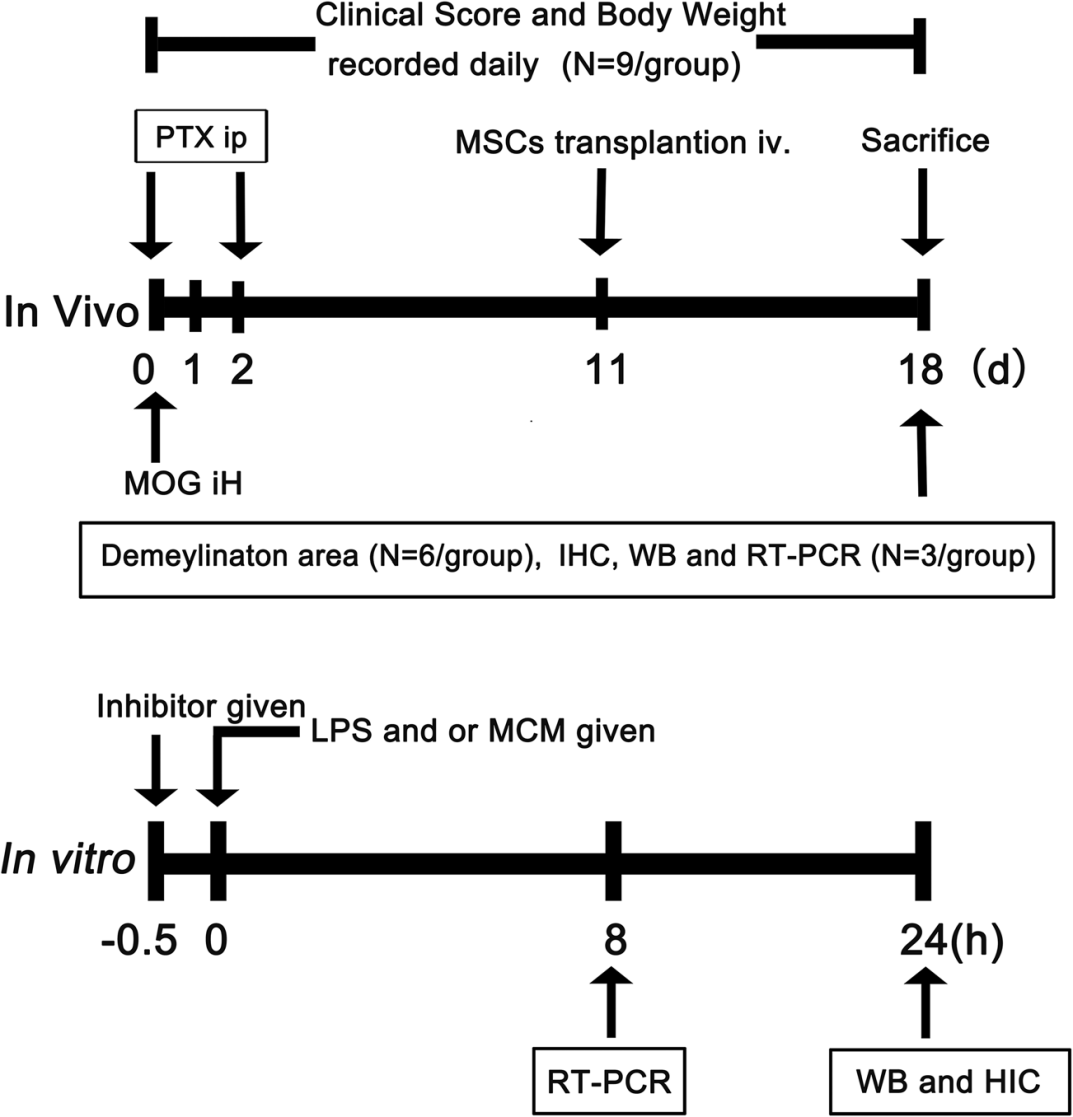
Experimental design. Graphs illustrate the experimental design both in vivo and in vitro. In the in vivo experiment, we transplant MSCs in EAE mice via tail vein at the day of disease onset. In the in vitro experiment, primary astrocytes are used, and the inflammatory reaction is induced by LPS. MCM mesenchymal stem cells conditioned medium, BAY BAY60-6583, a selective A_2B_AR agonist, SB SB203580, a p38 inhibitor

Animal protocol was approved by the Institutional Animal Care and Use Committee of Second Military Medical University, Shanghai, China. C57BL/6 mice were divided into two groups that either underwent MSCs or PBS treatment. MSCs were transplanted via tail vein at the time of disease onset that is at 11 days post immunization. For each mouse, 5 × 10^5^ mesenchymal stem cells resuspended in 0.2 ml PBS were administrated and the same volume of PBS was used for the PBS group. The dose was chosen as previously described [16]. Mice underwent none of the treatment were defined as control group. At 18 days after immunization, mice were sacrificed, and samples were collected for further study. The whole experimental design and the number of animals used in the study were displayed in Fig. 1.

### EAE Induction and Neurological Function Measurement

Female C57BL/6 mice, 6 to 8 weeks old, were used for EAE induction. In brief, mice were immunized with 200 μg MOG33–35 peptide (GL Biochem, Shanghai, China) in incomplete Freund’s adjuvant (Sigma-Aldrich, St. Louis, USA) containing 2.5 mg/ml *Mycobacterium tuberculosis* (BD Difco, Michigan, USA) by subcutaneous injection as previously described [16]. On the day of immunization and 48 h later, 200 ng Pertussis Toxin (Merck, Darmstadt, Germany) in 0.2 ml PBS was injected intraperitoneally. Weight and clinical score were recorded daily. Neurobehavioral assessments were conducted by an experimenter who was blind to the treatment conditions. Clinical score was evaluated using a 5-point clinical score [10]: (0) no clinical sign; (1) tail weakness; (2) hindlimb weakness; (3) complete hindlimb paralysis; (4) hindlimb paralysis and some forelimb weakness; and (5) moribund or dead.

### Histochemistry

For tissue preparation, mice were anesthetized and received intracardial perfusion with saline followed by 4% paraformaldehyde in PBS. Lumbar spinal cord was removed and fixed in the fixative (Servicebio, Wuhan, China). Four-micrometer-thick paraffin sections were prepared for further study. Slices were stained with hematoxylin and eosin (H&E, Sigma-Aldrich) to detect inflammatory infiltrates. To estimate the degree of demyelination in lumbar spinal cord, Luxol Fast Blue (LFB) staining was used. For LFB staining, slices of lumbar spinal cord were stained in 0.1% LFB solution (Sigma-Aldrich) for 16 h, washed in 95% alcohol, and then placed in 0.05% lithium carbonate. Eight areas in white matter from each slice were photographed. For analysis of HE staining, polynuclear cells were counted in each field by an investigator blind to the treatment group. The degree of demyelination was calculated as ratio of demyelination area to total white matter area as previously reported [29].

### IgG Staining

IgG staining was used to evaluate the permeability of BBB and was examined as previously described. Briefly, spinal cord slices were incubated with biotinylated antibody for 30 min, rinsed in PBS, and incubated with ABC reagent (Vector Labs, Burlingame, CA) for 30 min. The immunoreactivity was visualized using DAB (Vector Labs) staining, and the slices were counterstained with hematoxylin. Slices were examined and photographed for further analysis. Photographs were analyzed by Image Pro Plus 6.0 software (Media Cybernetics, Bethesda, MD) for mean integrated optical density (IOD) analysis.

### Western Blot Analysis

Samples from the whole spinal cord were lysed in RIPA (Millipore, Bedford, MA) supplemented with cocktail (Thermo, Waltham, MA), 1 mmol/L PMSF (Thermo), and phosphatase inhibitor (Thermo). For Western blots analysis, denatured samples containing the same amount of proteins were loaded onto the resolving gel (EpiZyme, Shanghai, China) for electrophoresis. Proteins were then transferred onto a nitrocellulose membrane (Whatman, Piscataway, NJ). The membrane was blocked with blocking buffer (EpiZyme) and then incubated with primary antibodies at the following dilution AQP4 (1:2000 dilution, Proteintech, Wuhan, China), A_2B_AR (1:2000 dilution, Abcam, MA, USA), ZO-1 (1:1000 dilution, Proteintech), Occludin (1:3000 dilution, Proteintech), p-p38 (1:1000 dilution, CST, MA, USA), p38 (1:1000 dilution, CST), β-actin (1:1000 dilution, Santa Cruz, Texas, USA) at 4 °C overnight respectively. The membrane was washed, incubated with the appropriate HRP-conjugated secondary antibody for 1 h, and then reacted with enhanced chemiluminescence substrate (Pierce, Rockford, IL). The results were recorded by Quantity One image software (Bio-Rad, Hercules, CA) and relative intensity was calculated using Gel-Pro Analyzer software (Media Cybernetics).

### Real-Time PCR

Total RNA from the whole spinal cord was extracted using TRIzol reagent (Invitrogen) and dissolved in RNA free water according to the manufacturer’s instructions. A universal 2-step RT-PCR cycling condition was used: 95 °C for 30 s followed by 40 cycles of 95 °C for 5 s and 60 °C for 31 s. mRNA levels were normalized to the endogenous control GAPDH expression and were calculated as fold change relative to the control group [30].

### Cell Culture and Treatment Protocol

#### Astrocyte Isolation and Culture

Primary astrocytes were isolated and cultured as previously described with modification [31]. In brief, cerebral cortexes were removed from the C57BL/6 mice at the stage of P0 and then digested by incubation with 0.25% trypsin (Hyclone) for 10 min at 37 °C. The reaction was stopped by removing the trypsin and washing the cortex with cold PBS for three times. The tissue was mechanically dissociated with a pipet to ensure full dissociation into single cells. The cells were diluted with complete medium (Dulbecco’s Modified Eagle’s Medium (DMEM), supplemented with 10% fetal bovine serum (Corning), 100 mg/ml streptomycin, 100 U/ml penicillin), and filtered through a 70-μm Nylon cell strainer (Corning Falcon). The cells were centrifuged at 800 rpm for 5 min. After removing the supernatant, the pellet was resuspended in complete medium and plated in six- or twelve-well plates at a density of 10^4^ cells/cm^2^. After cells were confluent, microglia were then removed by shaking at 220 rpm for 18 h on a horizontal orbital shaker. The purified cells were cultured for another 24 h and used for further experiment.

#### Experimental Protocol

MSCs from C57BL/6 mice were purchased from the American Type Culture Collection (ATCC, Virginia, USA), cells were cultured and expanded according to the manufacturer’s instructions, and P9 cells were used for the experiment. MSCs-conditioned medium (MCM) was collected after 48 h’ culture using complete medium ((Dulbecco’s Modified Eagle’s Medium (DMEM), supplemented with 10% fetal bovine serum (Corning), 100 mg/ml streptomycin, 100 U/ml penicillin) and frozen for further used. On the day of treatment, MCM was diluted with the same volume of complete medium and then used for study. MCM and LPS (100 μg/ml, Sigma) were added to each well at the same time and incubated 24 h for Western blot analysis and 8 h for RNA analysis. For inhibition and activation studies, p38 MAPK inhibitor (SB203580, Selleck Chemicals) and A_2B_ adenosine receptor (A_2B_AR) agonist (BAY60-6583, Sigma-Aldrich) were added at the concentration of 10 μM 30 min before MCM treatment. Untreated cells were used as a control group. The whole experimental design was displayed in Fig. 1.

### Immunofluorescent Staining

Astrocytes were mounted on cell slides and were fixed with 4% paraformaldehyde for 10 min and then incubated in PBS containing 0.1% tritonX-100 for 15 min at room temperature. After blocking with 10% BSA for 1 h, cells were incubated with anti-AQP4 antibody (1:500 dilution, proteintech) overnight at 4 °C. Followed by washing three times with PBS, the cells were incubated with fluorescence conjugated secondary antibodies for 1 h at room temperature. Slices were examined and photographed for further analysis. AQP4 staining was computed as mean integrated optical density (IOD).

### Statistical Analysis

Results were expressed as mean ± SD. For comparison between two groups, a Student’s *t* test was used to determined statistical significance. For comparison among multiple groups, statistical significance was evaluated using one-way ANOVA followed by a Student-Newman-Keuls test. Difference with *p* < 0.05 was considered statistically significant.

## Results

### MSCs Reduced Spinal Cord Demyelination and Improved Neurobehavioral Outcomes

**Fig. 2:**
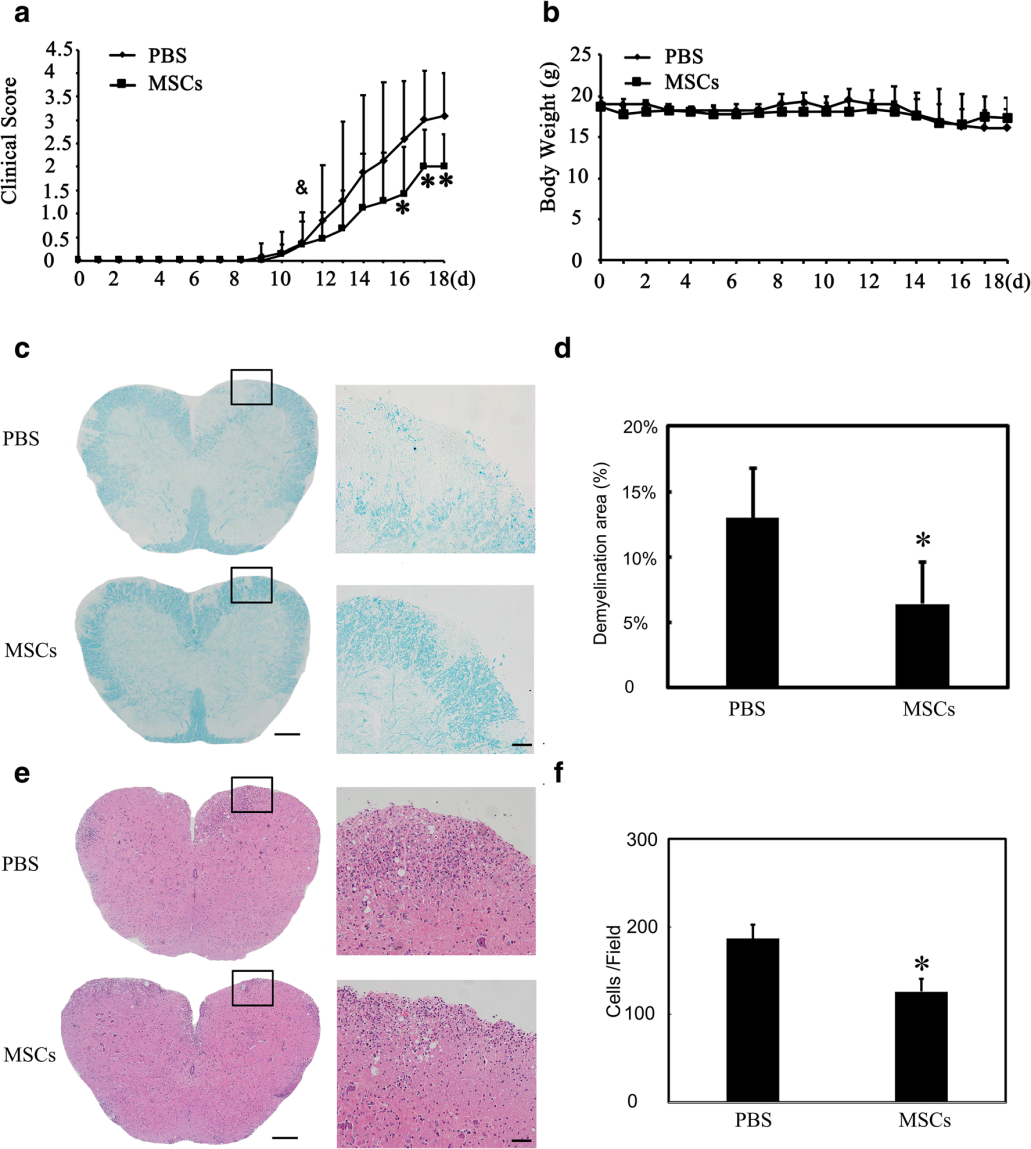
MSCs reduced spinal cord demyelination, neutrophil infiltration, and improved neurobehavioral outcomes in EAE mice. **a** Clinical score and **b** body weight are recorded daily in EAE mice in PBS- and MSCs-treated mice. *N* = 9 per group. **c** Photographs show LFB staining in PBS and MSCs-treated EAE mice. High magnification of the boxes is displayed at the right. Scale bar = 200 μm (left) and 50 μm (right). **d** Bar graph shows the percent of demyelination area in EAE mice (*n* = 6 per group). **e** Images show HE staining in EAE mice at 18 days post immunization (dpi) in the PBS and MSC-treated mice. High magnification of the boxes is displayed at the right. **f** Bar graph shows the number of inflammatory cells per field in EAE mice (*n* = 3 per group). Data are mean ± SD, *p < 0.05 MSCs vs. PBS group

Clinical score of EAE mice treated with MSCs or PBS was tested daily until 18 days post immunization (dpi) (Table 1). MSCs treatment improved neurobehavioral outcomes at 18 dpi (Fig. 2a, *p* < 0.05). MSCs administration had no effect on the body weight of the mice (Fig. 2b). LFB staining results indicated that MSCs treatment reduced spinal demyelination in EAE mice at 18 dpi (0.13 ± 0.04 vs 0.06 ± 0.03, Fig. 2c, d, *p* < 0.05). Further HE staining showed that MSCs administration alleviated neutrophil infiltration in the spinal cord of EAE mice (187 ± 16 vs 126 ± 15, Fig. 2e, f, *p* < 0.05).

**Table 1:** Clinical score of EAE mice in PBS and MSCs-treated EAE mice

Treatment	Number of mice	Average disease onset score	Average clinical score (16dpi)	Average clinical score (17dpi)	Average clinical score (18dpi)
PBS	9	0.4 ± 0.6	2.6 ± 1.2	3 ± 1	3.1 ± 0.9
MSCs	9	0.3 ± 0.5	1.4 ± 1*	2 ± 0.8*	2 ± 0.7*

The table shows the clinical score of EAE mice at disease onset, 16, 17, and 18 dpi in PBS and MSCs-treated mice. Data are mean ± SD. *p < 0.05 MSCs vs. PBS group

### MSCs Reduced BBB Disruption and IL-1β and TNF-α Expression in mRNA Level in EAE Mice

**Fig. 3:**
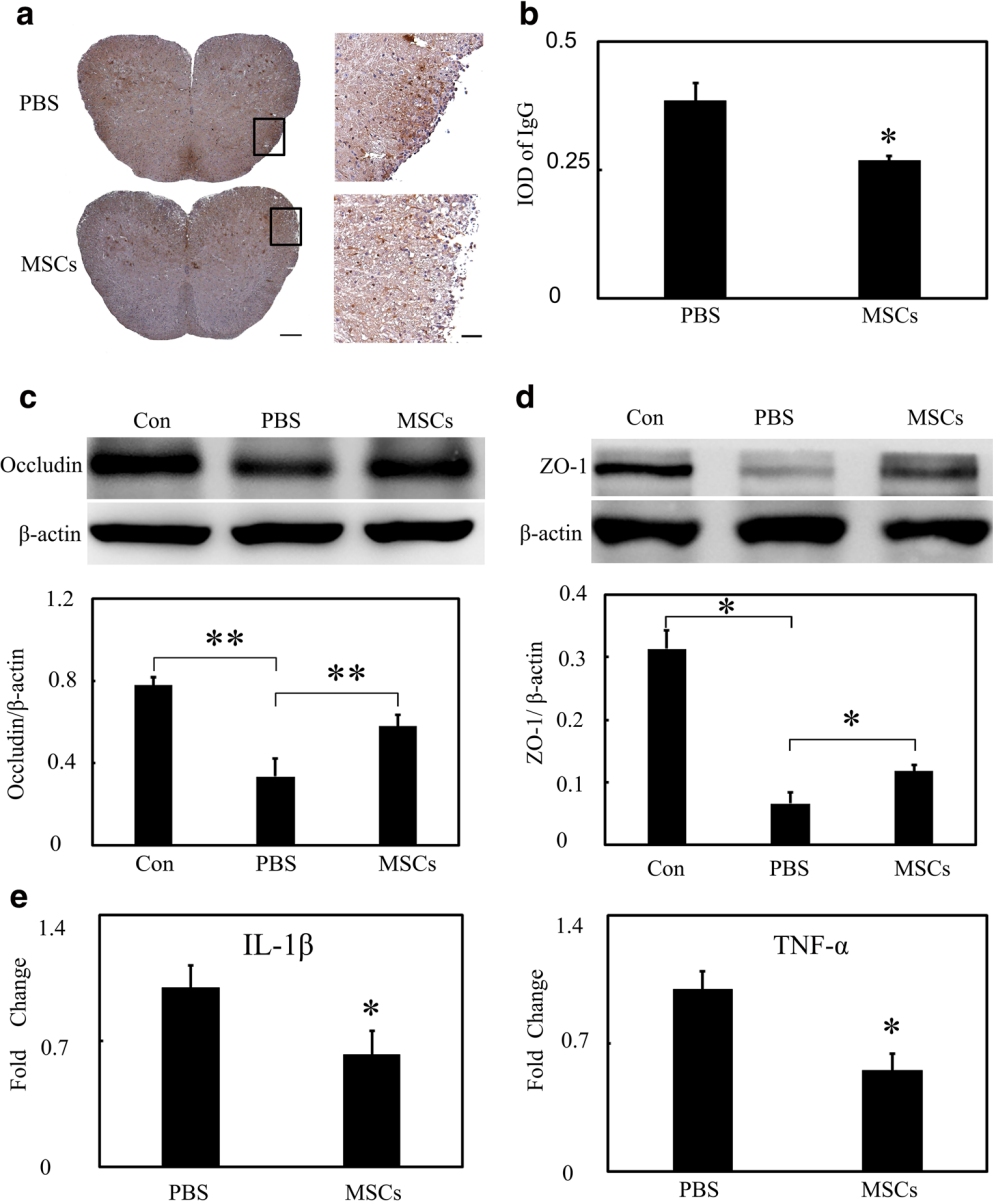
MSCs reduced IgG leakage and promoted ZO-1 and occludin rearrangement. **a** Photographs show IgG staining in PBS and MSCs-treated EAE mice. High magnification of the boxes is displayed at the right. Scale bar = 200 μm (left) and 50 μm (right). **b** Bar graph shows a semi-quantification of integrated optical density (IOD) in EAE mice (*n* = 3 per group). Representative results of occludin (**c**) and ZO-1 (**d**) expression at 18 dpi in EAE mice. Bar graphs show a quantification of occludin, ZO-1 expression (*n* = 3 per group). **e** Relative fold changes of inflammatory cytokine of IL-1*β* and TNF-α in PBS and MSCs-treated mice in EAE mice (*n* = 3 per group). Data are mean ± SD, *p < 0.05 MSCs vs. PBS group. **p < 0.01 MSCs vs. PBS group

To investigate the effect of MSCs on BBB permeability, we performed IgG immunostaining and found that MSCs reduced IgG leakage at 18 dpi (Fig. 3a, b, *p* < 0.05). Then, we conducted Western blot to evaluate tight junction expression. Results indicated that MSCs-treated mice had higher levels of occludin and ZO-1 expression (Fig. 3c, d, *p* < 0.05). RT-PCR revealed that the mRNA levels of inflammation-related cytokines including IL-1β and TNF-α were reduced after MSCs treatment compared with PBS treated mice (Fig. 3e, *p* < 0.05).

### MSCs Downregulated AQP4 and A_2B_AR Expression in EAE Mice

**Fig. 4:**
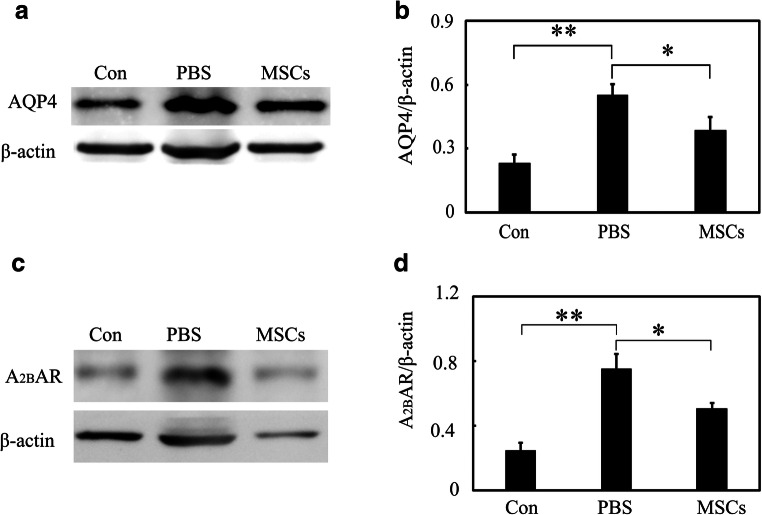
MSCs downregulated AQP4 and A_2B_AR expression in EAE mice. **a** Western blot of AQP4 expression in control, PBS, and MSCs group in EAE mice. **b** Bar graph shows a quantification of AQP4 (*n* = 3 per group). **c** Western blot of A_2B_AR expression in control, PBS, and MSCs group in EAE mice. **d** Bar graph shows a quantification of A_2B_AR (*n* = 3 per group). Data are mean ± SD, *p < 0.05 MSCs vs. PBS group. **p < 0.01 MSCs vs. PBS group

Western blot of AQP4 revealed that there was an increase in AQP4 expression in the spinal cord of EAE mice at 18 dpi. MSCs treatment significantly downregulated AQP4 expression (Fig. 4a, b, *p* < 0.05). In addition, we found that there was an upregulation of A_2B_ adenosine receptor (A_2B_AR) expression in EAE mice and MSCs treatment also reduced A_2B_AR expression (Fig. 4c, d, *p* < 0.05).

### MSCs Reduced AQP4 Expression in an A_2B_AR-p38 MAPK-Dependent Pathway

**Fig. 5:**
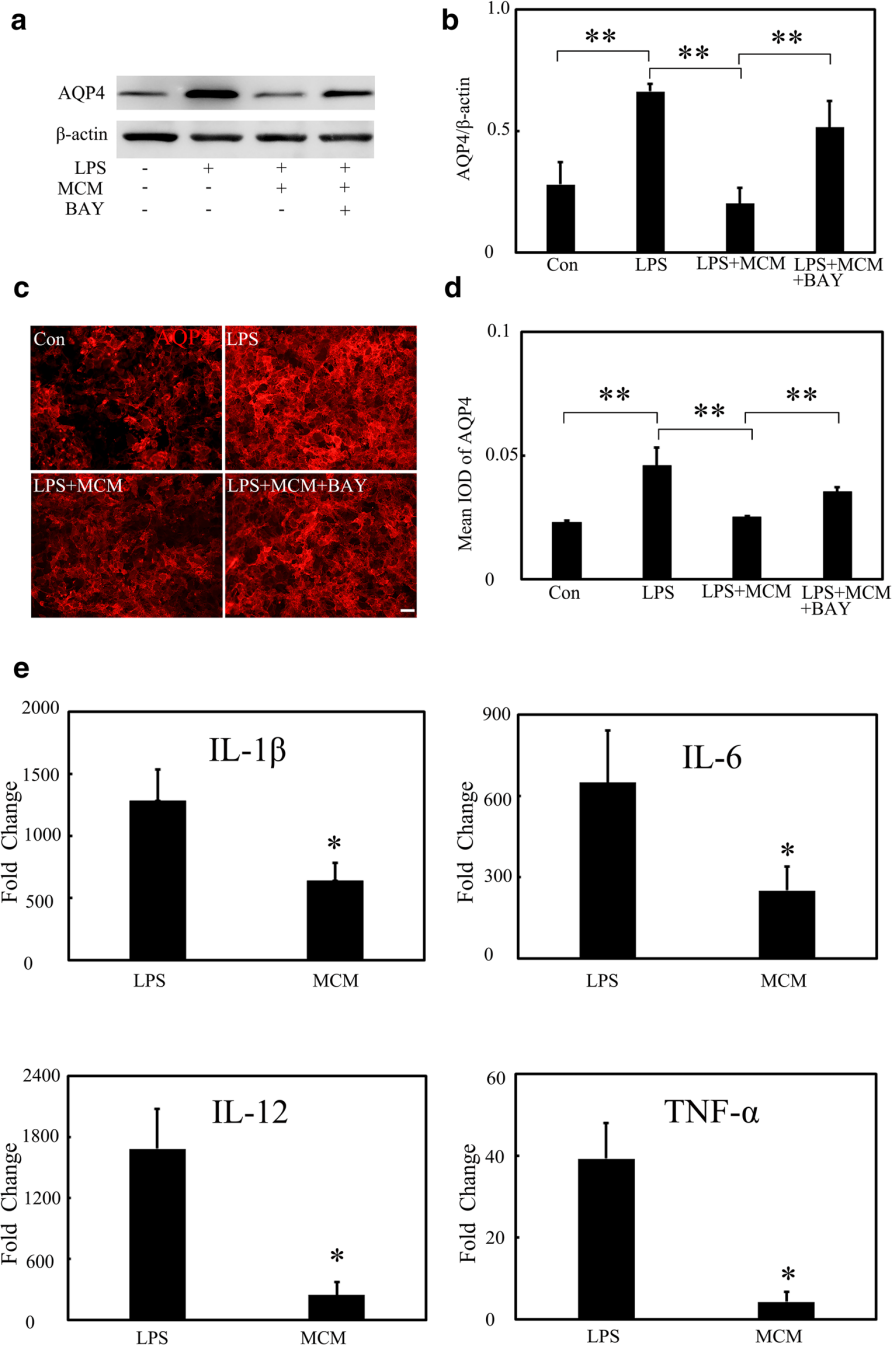
MSCs inhibited inflammatory cytokines release and downregulated AQP4 expression in astrocytes via inhibiting A_2B_AR expression. **a** AQP4 expression after LPS, MCM, and BAY treatment in primary culture astrocytes. **b** Bar graph shows a quantification of AQP4 expression. **c** Images show AQP4 staining in Con, LPS, LPS + MCM, LPS + MCM + BAY group. Scale bar = 50 μm. **d** Bar graph shows a semi-quantification of integrated optical density (IOD) of AQP4. **e** Relative fold changes of inflammatory cytokines of IL-1β, IL-6, IL-12, and TNF-α in LPS and MCM group. Data are mean ± SD, *p < 0.05, **p < 0.01. Representative results from three independent experiments are shown. MCM mesenchymal stem cells conditioned medium, BAY BAY60-6583, a selective A_2B_AR agonist

**Fig. 6:**
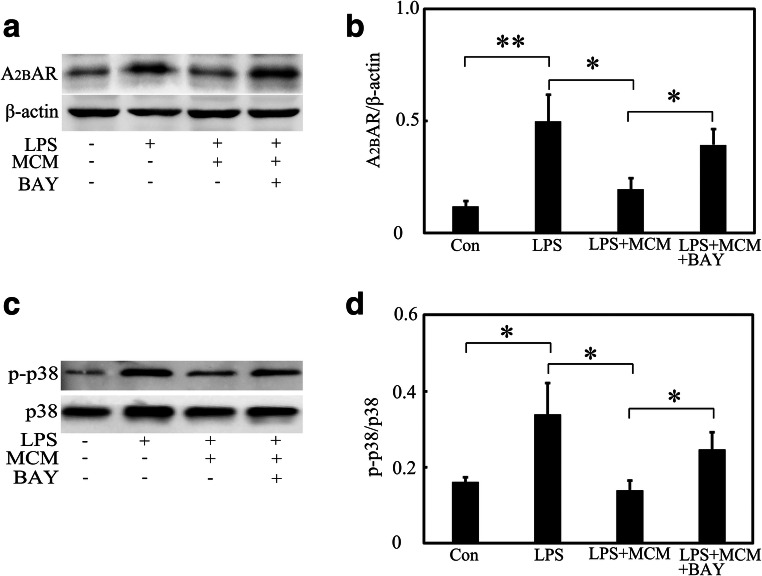
MSCs downregulated A_2B_AR and p38 MAPK expression in vitro. **a** A_2B_AR expression after LPS, MCM, and BAY treatment in primary astrocytes. **b** Bar graph shows a quantification of A_2B_AR expression. **c** p-p38 and p38 expression after LPS, MCM, and BAY treatment in primary astrocytes. **d** Bar graph shows a quantification of AQP4 expression. Data are mean ± SD, *p < 0.05, **p < 0.01. Representative results from three independent experiments are shown. MCM mesenchymal stem cells conditioned medium, BAY BAY60-6583, a selective A_2B_AR agonist

To determine whether MSCs downregulated AQP4 expression via A_2B_AR, we analyzed the effects of MSCs on AQP4 expression in primary culture of astrocytes. We treated astrocytes with LPS and/or MSCs-conditioned medium (MCM) to determine whether MCM could downregulate AQP4 expression. Using Western bolt, we found that LPS treatment increased AQP4 expression in astrocytes, MCM treatment reduced AQP4 expression in LPS-treated astrocytes (Fig. 5a, b, *p* < 0.01), and these results were verified by AQP4 immunostaining (Fig. 5c, d, *p* < 0.01). We also evaluated the effects of MSCs on astrocytic cytokine expression in mRNA levels. Results showed that MCM effectively reduced IL-1β, IL-6, IL-12, and TNF-α expression (Fig. 5e, *p* < 0.05). Then, we evaluated A_2B_AR expression under these tested conditions. After LPS treatment, there was an increase in A_2B_AR expression, and MCM treatment downregulated A_2B_AR expression at the protein level (Fig. 6a, b, *p* < 0.05).

**Fig. 7:**
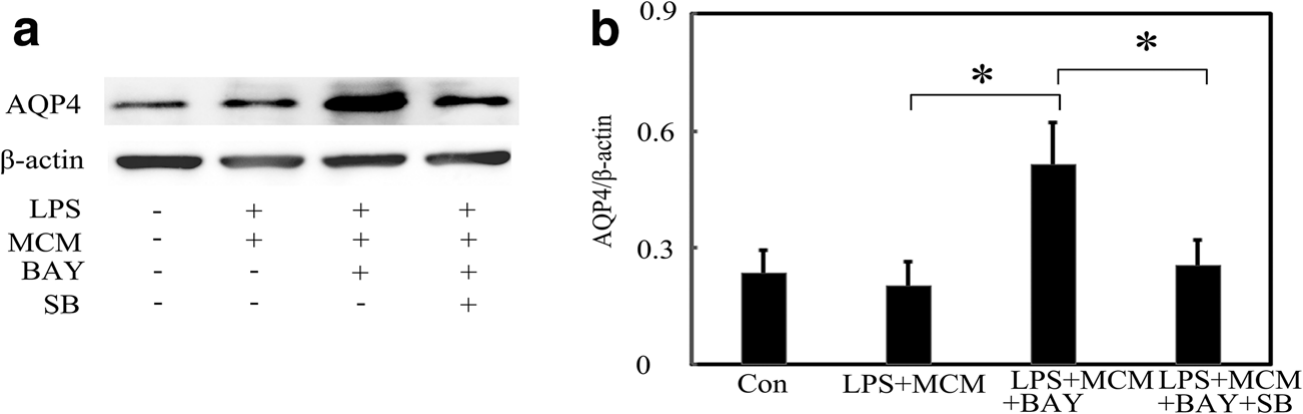
MSCs downregulated AQP4 expression via an A_2B_AR-p38 MAPK-dependent pathway in vitro. **a** AQP4 expression after LPS, MCM, BAY, and p38 inhibitor treatment in primary astrocytes. **b** Bar graph shows a quantification of AQP4 expression. Data are mean ± SD, *p < 0.05. Representative results from three independent experiments are shown. MCM mesenchymal stem cells conditioned medium, BAY BAY60-6583, a selective A_2B_AR agonist. SB=SB203580, a p38 inhibitor

To determine whether A_2B_AR signaling pathway was involved in AQP4 expression, A_2B_AR agonist BAY60-6583 was used. Results showed that BAY60-6583 upregulated A_2B_AR expression in astrocytes compared with the LPS + MCM group (Fig. 6a, b, *p* < 0.05), and this parallels to an increased expression of AQP4 (Fig. 5a, b). Furthermore, we used Western blot to evaluate the effects of MSCs on p38 MAPK phosphorylation. The results indicated that MCM inhibited LPS-induced p38 MAPK activation in astrocytes and this effect was reversed by BAY60-6583 (Fig. 6c, d, *p* < 0.05). At last, we used p38 MAPK inhibitor SB203580 to evaluate the relationship between p38 MAPK and AQP4. As it has been shown in Fig. 5a, b, LPS treatment increased AQP4 expression in astrocytes and MCM treatment reduced LPS-induced upregulation of AQP4 expression. Compared with LPS + MCM + BAY60-6583 group, when SB203580 was added, AQP4 expression was again decreased (Fig. 7a, b, *p* < 0.05). Thus, we concluded that MSCs reduced AQP4 expression via A_2B_AR-p38 MAPK-mediated pathway.

## Discussion

In this study, we found that bone marrow MSCs exerted therapeutic effects via maintaining the integrity of BBB and downregulating AQP4 expression in EAE mice, contributing to reduced demyelination and inflammatory cell infiltration in the spinal cord. In addition, we found that A_2B_AR agonist, BAY-60-6583, reversed MSCs-induced downregulation of AQP4 expression in cultured astrocytes. Thus, we concluded that bone marrow MSCs exerted its protective effects partly through downregulating AQP4 expression in MS.

The disturbance of BBB was the first pathological process in MS, which was ahead of demyelination [6]. BBB disruption directly resulted in inflammatory cell infiltration, which subsequently activated the immune reaction in the central nervous system. Therapies targeting reducing tight junction degradation maintained BBB integrity and limited lymphocyte infiltration during inflammation [5]. Protease activated receptor-1 (PAR-1) antagonist maintained the integrity of BBB and attenuated the clinical symptoms of EAE mice effectively [32]. Increasing PRDX6 expression in astrocytes of EAE mice could reduce BBB disruption and clinical severity [7]. The same result was also found in mice overexpression of claudin-1 [6]. Previous studies demonstrated that only MSCs administration before or at the disease onset could it exert protective effects [10, 16], which indicated MSCs might affect BBB integrity or infiltration of inflammatory cells. In this study, we demonstrated that MSCs administration at the disease onset maintained BBB integrity in EAE mice. We also found that MSCs reduced inflammatory expression in mRNA level in the spinal cord of EAE mice, which might be due to maintain BBB integrity.

Although the effects of MSCs in MS have been widely investigated, effect of MSCs on BBB integrity in MS is still unclear. In this research, we demonstrated that the beneficial effects of MSCs on BBB attributed to reduced AQP4 expression. In transient middle cerebral artery occlusion (tMCAO) mice model, MSCs transplantation effectively downregulated AQP4 expression and reduced astrocyte apoptosis, thus maintaining the integrity of BBB [18]. Downregulating AQP4 expression reduced the disruption of BBB in tMACO mice [28]. Thus, we speculated that the downregulation of AQP4 expression might protect from the disruption of BBB. In both human MS lesion and EAE mice, the increased expression of AQP4 has been observed [25, 26]. In our study, we found increased expression of AQP4 in the spinal cord of EAE mice and in LPS-activated astrocytes in vitro. In AQP4 deficiency EAE mice, neurological impairment was alleviated, which indicated that AQP4 is a promising therapy target in MS. Methods downregulating AQP4 expression might be effective. In consistent with our speculation, we found that downregulating AQP4 expression by MSCs administration effectively protected the EAE mice from inflammation-induced injury. Collectively, these results suggested that MSCs could exert protective effects in EAE mice via downregulating AQP4 expression in MS.

The mechanism by which MSCs downregulated AQP4 expression has not been fully illustrated. Our previous study found that MSCs reduced AQP4 expression in astrocytes through regulating p38 MAPK pathway [18]. In the research, we found that MCM downregulated AQP4 expression and this effect was reversed when BAY-60-6583, an A_2B_ AR agonist was added. Thus, we speculated that A_2B_AR was also participated in the regulation of AQP4 expression. A_2B_AR is a low affinity receptor for adenosine and is activated under trauma, inflammation, ischemia, or other types of stressful insults [33]. It plays a pro-inflammatory role in human asthma and chronic obstructive pulmonary disease and murine colitis [34]. A_2B_ AR has been shown to regulate p38 MAPK signaling pathway in astrocytes and in EAE mice. In ischemic conditions, A_2B_AR/p38 MAPK signaling pathway participated in the inhibition of ceramide production in astrocytes [35]. In EAE mice, both A_2B_AR antagonists and A_2B_ AR-knockout alleviated the clinical symptoms and blocked IL-6 production. The p38 MAPK pathway was involved in the A_2B_AR-mediated IL-6 production [36]. Thus, we speculated that MSCs reduced AQP4 expression via A_2B_AR-p38 MAPK pathway. In EAE mice, we found there was an increase in A_2B_ AR expression in the spinal cord at 18 dpi in protein level, and MSCs treatment reduced A_2B_AR expression. In our in vitro study, MSCs reduced A_2B_AR expression in primary astrocytes and A_2B_AR agonist BAY-60-6583 reversed MSCs-induced downregulation of AQP4 and promoted phosphorylation of p38 MAPK. Therefore, we conclude that MSCs maintained BBB integrity in EAE mice via downregulating AQP4 expression in an A_2B_AR-p38 MAPK-dependent manner.

## Conclusions

We demonstrated for the first time that MSCs downregulated AQP4 expression in EAE mice, which consequently attenuated inflammation-induced BBB disruption and contributed to improved clinical outcomes. A_2B_AR-p38 MAPK signal pathway was associated with this regulation effects. Our study provided a new insight on the mechanism of MSCs therapy in MS.

## Data Availability

The datasets generated for this study are available on request to the corresponding author.
